# Towards Zero Training for Brain-Computer Interfacing

**DOI:** 10.1371/journal.pone.0002967

**Published:** 2008-08-13

**Authors:** Matthias Krauledat, Michael Tangermann, Benjamin Blankertz, Klaus-Robert Müller

**Affiliations:** Machine Learning Laboratory, Berlin Institute of Technology, Berlin, Germany; Indiana University, United States of America

## Abstract

Electroencephalogram (EEG) signals are highly subject-specific and vary considerably even between recording sessions of the *same user* within the same experimental paradigm. This challenges a stable operation of Brain-Computer Interface (BCI) systems. The classical approach is to train users by neurofeedback to produce fixed stereotypical patterns of brain activity. In the machine learning approach, a widely adapted method for dealing with those variances is to record a so called calibration measurement on the beginning of each session in order to optimize spatial filters and classifiers specifically for each subject and each day. This adaptation of the system to the individual brain signature of each user relieves from the need of extensive user training. In this paper we suggest a new method that overcomes the requirement of these time-consuming calibration recordings for long-term BCI users. The method takes advantage of knowledge collected in previous sessions: By a novel technique, prototypical spatial filters are determined which have better generalization properties compared to single-session filters. In particular, they can be used in follow-up sessions without the need to recalibrate the system. This way the calibration periods can be dramatically shortened or even completely omitted for these ‘experienced’ BCI users. The feasibility of our novel approach is demonstrated with a series of online BCI experiments. Although performed without any calibration measurement at all, no loss of classification performance was observed.

## Introduction

A Brain-Computer Interface (BCI) based on electroencephalogram (EEG) signals provides a direct communication channel for healthy or disabled users from the brain to a technical device. Through motor imagery or movement intentions brain activity can be voluntarily modulated in a predictable way. A BCI system can detect these alterations in the ongoing EEG and control an application (text-entry system; prosthesis; computer game) accordingly. Since no peripheral nerves or muscles need to be involved in this process, BCI technology may be used in assistive technology for paralyzed patients. One classical approach to establish EEG-based control is to set up a system that is controlled by a specific EEG feature which is known to be susceptible to conditioning and to let the subjects learn the voluntary control of that feature in a learning process that can last several weeks. In contrast, in the machine learning approach to BCI [1], [2] a statistical analysis of a calibration measurement which is recorded at the beginning of each session is used to adapt the system to the specificities of the user's current brain signals. This approach allows for an effective performance from the first session on without user training [3], [2]. As the signals vary between sessions even for the same user, machine learning based BCI systems rely on the calibration procedure for optimal performance (machine training).

To present, the use of machine learning based EEG-BCI systems involves two time-consuming preparational steps at the beginning of every new session. The first one, the montage of an EEG cap, has been largely alleviated by recent hardware advancements (see [4] and the discussion section of this paper). The second step is the recording of calibration data, which we will address with this *online* study.

Especially for patients with impaired concentration ability, this initial calibration reduces the valuable remaining time for controlling a device or computer software in the so called feedback application phase. But even for healthy users, the calibration is an annoying procedure.

In an offline study, Krauledat et al. [5] recently proposed a new method for avoiding subject training under conditions that could easily be met in practice.

The basic idea of the method is as follows: In the case of long-term BCI users, who repeatedly perform BCI sessions with the same mental tasks, one can exploit data from previous sessions in order to learn most of the calibration parameters. This saves time in the setup of the next session.

**Figure 1:**
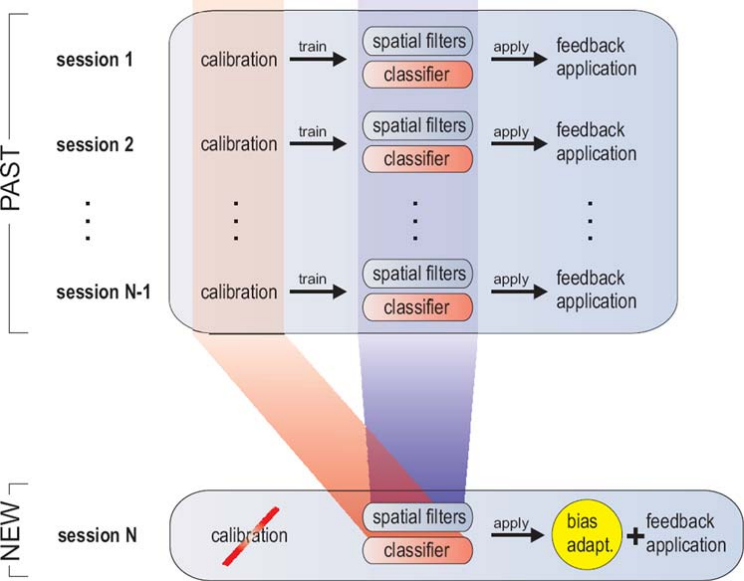
Sessions *1* to *N-1* show a standard BCI procedure: spatial filter and classifiers are learned each session anew from a calibration recording (e.g. with CSP and LDA) before they are applied during a feedback application. The new *Zero-Training* method eliminates the calibration recording: spatial filters and a classifier are predetermined before session *N* starts. The spatial filters for session *N* are extracted from old spatial filters (blue), the classifier for session *N* is calculated from old calibration recordings (red). The feedback application of session *N* is preceded only by a very quick bias adaptation (yellow).

The present study now extends the offline study in [5] by an online application and evaluation, which will further be called the *Zero-Training* method. In more detail, we show how to learn good spatial filters and classifiers from data of previous sessions which eliminates the necessity of going through a new phase during each new session (see Figure 1). The method is tested against the standard approach where spatial filters and classifiers are trained anew on the calibration data of a new session.

The structure of the paper is the following: In the first subsection of the Methods-section, the common spatial pattern (CSP) method is explained in detail, as CSP is important for our proposed new *Zero-Training* method. The second subsection shows how so-called prototype patterns can be extracted from previous BCI sessions and how a classifier can be prepared in advance of a new BCI session. In the following, we introduce an experimental setting that allows for the comparison of the *Zero-Training* approach with the ordinary approach including calibration. Finally, we show the results of this comparison, discuss our findings and end with a conclusion.

## Methods

### 1. Background

#### A. Neurophysiology

Macroscopic brain activity during resting wakefulness contains distinct ‘idle’ rhythms located over various brain areas. Sensorimotor cortices show rhythmic macroscopic EEG oscillations (μ-rhythm or sensorimotor rhythm, SMR), with spectral peak energies of about 8–14 Hz (α-band) and/or 16–28 Hz (β-band) localized in somatosensory cortex [6].

A large class of EEG-based BCI systems relies on the fact that amplitude modulations of sensorimotor rhythms can be caused, e.g. by imagining movements. For example, the power of the μ-rhythm decreases during imagined hand movements in the corresponding representation area which is located in the contralateral sensorimotor cortex. This phenomenon is called event-related desynchronization (ERD, [7], [8]), while the increase of band power is termed event-related synchronization (ERS). This may be observed, e.g., during motor imagery over flanking sensorimotor areas, possibly reflecting an ‘surround inhibition’ enhancing focal cortical activation, see [9], [8]. The exact location and the exact frequency band of the sensorimotor rhythm is subject-specific. Hence individually optimized filters can increase the signal-to-noise ratio dramatically [10]. To this end, the CSP technique has proven to be useful.

#### B. Common Spatial Pattern (CSP) Analysis

Common Spatial Pattern and its extensions (e.g. [11], [12], [13], [14], [10]) is a technique to analyze multi-channel data based on recordings from two classes (conditions). It is, e.g. used in BCI systems based on the modulation of brain rhythms. CSP filters maximize the EEG signal's variance under one condition while simultaneously minimizing it for the other condition. Since variance of band-pass filtered signals is equal to band power, CSP analysis is applied to band-pass filtered signals in order to obtain an effective discrimination of mental states that are characterized by ERD/ERS effects (see above). In the example of left vs. right hand motor imagery, the CSP algorithm will find two groups of spatial filters. The first will show high band power during left hand motor imagery and low band power during right hand motor imagery, and the second vice versa.

Let Σ*_i_* be the covariance matrix of the trial-concatenated matrix of dimension [*C*×*T*] (where *C* is the number of electrodes and *T* is the number of concatenated samples) belonging to the respective class *i*∈{1,2}. The CSP analysis consists of calculating a matrix 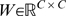 and a diagonal matrix *D* with elements in [0,1] such that
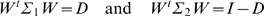
(1)where 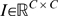 is the identity matrix. This can be solved as a generalized eigenvalue problem. The projection that is given by the *i*-th column of matrix *W* has a relative variance of *d_i_* (*i*-th element of *D*) for trials of class 1 and relative variance 1−*d_i_* for trials of class 2. If *d_i_* is near 1, the filter given by the *i*-th column of *W* (i.e., the *i*th spatial filter) maximizes the variance for class 1, and since 1−*d_i_* is near 0, it also minimizes the variance for class 2. Typically one would retain projections corresponding to two or three of the highest eigenvalues *d_i_*, i.e., CSP filters for class 1, and projections corresponding to the two or three lowest eigenvalues, i.e., CSP filters for class 2.

For a detailed review of the CSP technique with respect to the application in BCI see [10].

#### C. Features and Classification

He were describe generally, how spatial CSP filters are used to calculate features for classification, and how the ongoing EEG is translated into a control signal. This method applies to both classical CSP and the proposed method.

The EEG signals of the calibration measurement are band-pass filtered (subject-specific frequency band, see Section “Experimental Setup” and Table 1) and spatially filtered with the selected CSP filters. From these signals the log-variance is calculated in each trial of the calibration data (interval is selected subject-specifically, typically 750 to 3500 ms relative to the presentation of the visual cue). This procedure results in a feature vector with dimensionality equal to the number of selected CSP filters (which was in this study 4 for classical CSP and 12 for the proposed method, see Section “Construction of Classifiers”). For classification least squares regression (LSR) was used.

**Table 1:** Subject-specific parameters.

Subject	#channels	#past sessions	#train trials	Classes	FQ band		Interval	
					(CSP)	(*ZT*)	(CSP)	(*ZT*)
*zq*	46	7	845	LR	[9 14]	[9 25]	[810 4460]	[500 3000]
*ay*	46	4	324	LR	[8 22]	[9 25]	[710 2650]	[500 3000]
*zp*	46	5	704	LR	[10 25]	[9 25]	[2750 5000]	[500 3000]
*al*	44	9	684	FR	[11 25]	[9 25]	[1600 4690]	[500 3000]
*aw*	44	13	1075	LF	[11 17]	[10 25]	[1500 4500]	[500 3000]
*zk*	46	7	240	LR	[8 31]	[9 25]	[920 4390]	[500 3000]

The first until third column report the number of sensors and sessions, as well as the number of trials per class which were available in total from these previous sessions. The fourth column indicates the two motor imagery classes that have been used (L: left hand, R: right hand; F: right foot). The frequency band (FQ band) for CSP analysis was chosen for each subject individually. For original CSP (column 5) it was chosen on data of the actual session. For *Zero-Training (ZT)* (column 6) it was chosen on data from previously available sessions. The same holds for the time window used for the training of the classifier, denoted in milliseconds after stimulus presentation: for CSP (column 7), the window was optimized on the training data, while for *Zero-Training*, a fixed window was used for all subjects.

For online operation, features are calculated in the same way every 40 ms from the most recent segment of EEG (sliding windows of 1000 ms width). CSP filters calculated from the initial calibration measurement are not adapted during online operation. Nevertheless the system allows stable performance even for several hours [15], [16]. But for optimal feedback the bias of the classifier might need to be adjusted for feedback. Since the mental state of the user is very much different during the feedback phase compared to the calibration phase, also the non task related brain activity differs. For a thorough investigation of this issue cf. [17], [18], [19]. With regard to this study, the issue is discussed in Section “Experimental Setup”.

#### D. Preliminary Study

In [5], we have analyzed data from the same subjects in repeated BCI sessions, that were recorded with the same motor imagery paradigms. We could show that the proposed distance (which will be introduced in detail in Section “Prototype Filters”) clearly groups corresponding spatial filters into clusters, and the clusters themselves could be interpreted as physiologically relevant groups of filters. We used this concept to extract prototypical filters from previous sessions of a particular subject. In an offline analysis, it could be shown that the proposed method outperforms the usual CSP routine even if the number of training samples for CSP is increased up to 30 trials per class from the same session.

The encouraging result was that high classification performance for longterm BCI users can be established with no or very few calibration trials from the current session. In the current work, we expand this finding to the online scenario.

### 2. Prototype Filters

The CSP filters are not just randomly drawn points from 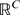, but instead represent subject-specific neurophysiological conditions, which suggests that, for a given subject, similar filters should be found across all sessions. We will first define a meaningful notion of similarity in this space and then use this relation to explore the space. We expect that the regions with a high density of CSP filters contain examples for filters which are particularly stable and informative across sessions. We will call these regions “clusters”, and we will introduce a method how to sample prototypical filters from the clusters, using a notion of “inlier” points which have a low distance to their nearest neighbors [20], [21].

#### A. Metric in the Space of CSP Filters and γ-Index

**Figure 2:**
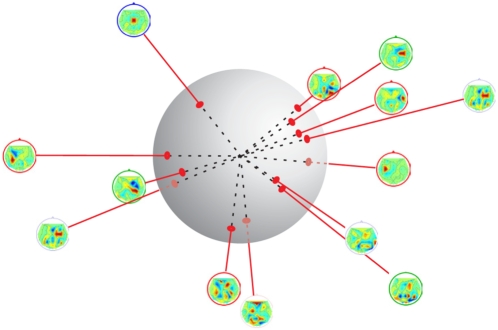
Projection of CSP filters onto the (*C*−1)-dimensional hypersphere. Distances between filters are defined by the angles between the projected filters.

CSP filters are obtained as solutions of a generalized eigenvalue problem. Since every multiple of an eigenvector is again a solution to the eigenvalue problem every point in the space of CSP filters (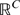) on the line through a CSP filter point and the origin form an equivalence class (except for the origin itself). More precisely, it is sufficient to consider only normalized CSP vectors on the (*C*−1)-dimensional hypersphere (cf. figure 2).

This suggests that the CSP space is inherently non-euclidean. As a more appropriate metric between two points *w*_1_ and *w*_2_ (column vectors of a CSP filter matrix *W*) in this space, we calculate the angle between the two lines corresponding to these points:
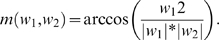
(2)When applying this measure to a set of CSP filters (*w_i_*)*_i_*_≤*n*_, one can generate the distances (*m*(*w_i_*, *w_j_*))*_i_*_,*j*≤*n*_, which can then be used to find prototypical examples of CSP filters.

Once a suitable distance function is established, it can be used to find regions in the data space consisting of CSP filters, which are more densely sampled than others (‘clusters’). In particular, by identifying points located in the middle of clusters, it is possible to select them as typical CSP filters. We apply a clustering concept which has been introduced by [21] and consider the average distance (according to metric *m*) of a filter to its *k* = 5 nearest neighbors.

Let nn_1_(*w*),…,nn*_k_*(*w*) be the *k* nearest neighbors to point *w* according to metric *m* (Eq. (2)). Then the average distance of *w* to its neighbors is called the γ-index of *w*, i.e.
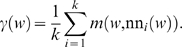
The CSP filter with the lowest γ-index can clearly be regarded as “inlier”-point of a cluster. In order to find other regions of the filter space which are also densely populated, we applied a heuristic which is presented in the next section.

#### B. Finding Cluster Prototypes

We first calculated the γ-index of each filter to obtain a ranking according to the distance function explained above. The lowest γ-index indicates that the corresponding filter is inside a region with many other filter examples and should therefore be chosen as cluster prototype. The same applies to the second-to-lowest γ-index, but in this case it would not be recommendable to select this filter, since it is highly probable that the filter is from the same region as the first one. To ensure that we also sample prototypes from other clusters, an incremental procedure of choosing and re-weighting is applied to determine a predefined number of cluster prototype filters.

The search starts with one prototype only, that is chosen as the filter with the minimal overall γ-index. The chosen filter point is removed from the set of all filter points. Then the average distance of each remaining filter to its neighbors is re-weighted by the inverse of the distance to the removed point, as explained in [21]. Due to this re-weighting, all points in the vicinity of the chosen cluster prototype receive a larger γ-index. The re-weighting is driven by the assumption that these neighboring points belong to the same cluster with high probability. Due to their increased γ-index, they are less likely chosen as prototypes in the next iteration. The iterative procedure ends, when a predefined number of cluster prototypes has been determined.

### 4. Experimental Setup

To demonstrate the feasibility of the *Zero-Training* approach, a BCI feedback study was designed to compare the proposed approach with the classical CSP approach in terms of feedback performance. The specific construction of the two classification setups is described in Section “Construction of Classifiers”.

The BCI experiments were performed with 6 healthy subjects, 5 male and one female, aged 26–41. These were all the subjects who previously had performed at least 5 BCI sessions with the Berlin Brain-Computer Interface (BBCI). They were members of the department and volunteered for the participation in this study. The availability of a large amount of experimental data is a prerequisite for the extraction of prototypical CSP filters as described in Section “Prototype Filters”, since the cluster density in the CSP filter space can only be estimated reliably with a sufficient number of sample points.

**Figure 3:**
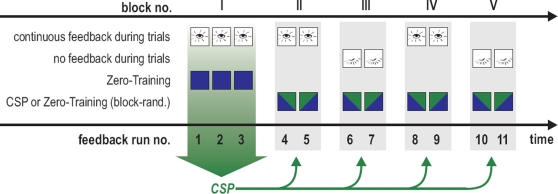
Overview of the 11 runs and the two methods used for calculating feedback. In block I, continuous visual feedback was given by a classifier that had been pre-computed with the *Zero-Training* method, see Section “Construction of Classifiers”. Blocks II to V each contained one run with *Zero-Training* feedback and one run with ordinary CSP feedback. The order of the two feedback methods was chosen randomly and remained unknown to the subject. The use of continuous visual feedback and no continuous visual feedback alternated regularly between blocks II to V.

The visual feedback consisted of the presentation of a computer cursor which was controlled by the output of one of two different classifiers. The goal of each trial was to steer the computer cursor in eleven feedback runs that were grouped in five experimental blocks (see Figure 3). In block I, continuous visual feedback was given by a classifier that had been pre-computed with the *Zero-Training* method, see Section “Construction of Classifiers”. Data collected in these initial three runs were used to determine spatial filters and a classifier using the ordinary CSP method (as described in Section “Construction of Classifiers”) for use in the following blocks. Blocks II to V each contained one run with *Zero-Training* feedback and one run with CSP feedback. Within a block, the order of the two feedback methods was chosen randomly and remained unknown to the subject. The use of continuous visual feedback and no continuous visual feedback alternated regularly between blocks II to V, as indicated in Figure 3.

During the experiment the subjects were sitting in a comfortable chair in front of a computer screen. EEG was recorded with 64 Ag/AgCl electrodes, acquired at a sampling rate of 1000 Hz, then downsampled to 100 Hz. The resulting data was bandpass-filtered at a subject-specific frequency band (see Table 1), and spatial filters, as described in Sections “Prototype Filters” and “Common Spatial Patterns Analysis”, were applied. Finally, the logarithmic band power of the spatially and temporally filtered signals was estimated by calculating the logarithm of the squared sum of the filter outputs. These features were fed into a linear classifier. We used least squares regression (LSR), in order to force the classwise mean of the linear classifier output to be +1 and −1, respectively.

At a rate of 25 Hz, graded classifier outputs were calculated for the last 1000 ms, and averaged over 8 samples. A scalar factor was multiplied to the result, and finally a real-valued bias term was added.

Guided by our experience with non-stationary bias, a bias adaptation was performed at the beginning of every run. Therefore, the subject controlled the cursor for 20 trials (10 per class), and the bias was adapted at the end of this period. The procedure corresponds to the initial calibration of the bias as presented in [18].

In the following 100 trials (50 per class), the subject controlled the cursor in a feedback application. At the beginning of each trial, one of two boxes on either side of the screen was visually highlighted to indicate a new target. After being fixed in the middle of the screen for 750 ms, the cursor was released (see also the description in [2]). During these 3.5 seconds, the subjects were instructed to now imagine the associated motor movement (see Table 1 for the imagery used by each subject), in order to hit the target with the cursor. Depending on the block (see Figure 3), the cursor was either visible during this cursor movement phase (continuous visual feedback) or a blank screen was presented for 3.5 seconds (no continuous visual feedback).

The graded classifier output was used to control the cursor position in horizontal direction in a rate-controlled manner. After 3.5 seconds, the cursor was fixed again and the outcome of the trial was determined by the horizontal position of the cursor. If the cursor was on the correct side of the screen, the trial was counted as “hit”, and as “missed” otherwise. The target box was then colored according to the trial outcome in green (for a successful trial) or red (in the other case). The highlighting of the target box at the end of the trial was visualized again for all trials, that means also for blocks where no continuous visual feedback was given. After a short intertrial break of 1 second the next target was presented.

### 4. Construction of Classifiers

The following two sections describe how the spatial filters and classifier are determined for the proposed new approach and for the classical CSP approach.

The feedback performance of these two approaches is compared using the experimental design described in Section “Experimental Setup”. It will become clear, that both approaches will only use a small number of spatial filters (two or three per class) from the total set of filters provided by CSP. Although many more could be chosen in theory, experience with CSP for motor imagery paradigms has shown that further filters often model the noise of the data rather than the signals of interest. Thus the restriction to a small number of filters per class is helpful [10]. For a detailed discussion of the influence of data dimensionality on classification results please refer to the “Discussion” section.

#### A. The Zero-Training Filters and Classifier

For each subject, data from a number of past sessions (past data) is available (see Table 1). Based on this data, a set of spatial filters and the *Zero-Training* classifier is constructed individually for each subject. This preparation could take place days before the planned feedback experiment, as only historic data is involved for the construction of *Zero-Training*. For every subject, we performed the following:

For each class and for each historic session of the subject, we calculated the three filters with the largest eigenvalues using the CSP algorithm presented in Section “Common Spatial Patterns Analysis”. Depending on the number of past sessions, this procedure creates a larger set of filters.

Once this set of historic filters is created, 6 so-called *prototype filters* are chosen from the set applying the clustering method described in Section “Prototype Filters”. Those filters constitute the first 6 dimensions of the final feature space for the *Zero-Training* method. In addition to these prototypical filters, we also pool all the data from past experiments of the subject of interest and calculated the ordinary CSP filters on this collection of historic data sets. The resulting CSP filters (3 per class) are concatenated to the 6 prototype filters gained from the clustering approach.

With this approach, filtering the EEG data of the pooled data set (all past sessions of the subject) results in a 12-dimensional feature space. Finally, a linear classifier is calculated on the features using Least Squares Regression (LSR). If necessary we could also use nonlinear classification here (cf. [22], [23]).

#### B. The Ordinary CSP Filters and Classifier

For each subject, we also build a set of ordinary CSP filters and a corresponding classifier. In contrast to the *Zero-Training* solution, they can not be prepared beforehand. Their construction is done on the fly during a new experimental session and does not involve data from past sessions.

For the training of a regular CSP classifier, we first record three runs of feedback data (with feedback provided by the output of the *Zero-Training* classifier), totalling to more than 150 trials per class. According to the cross-validation error on this data, the optimal frequency band is selected, as well as some additional parameters like length and starting point of the training time interval for estimating the band power. The Common Spatial Patterns are computed on this data and the two spatial filters representing the most extreme eigenvalues are chosen for each class.

Then a linear classifier (LSR) was trained using the preprocessed data from the first three runs.

## Results

### A. Feedback Performance

**Figure 4:**
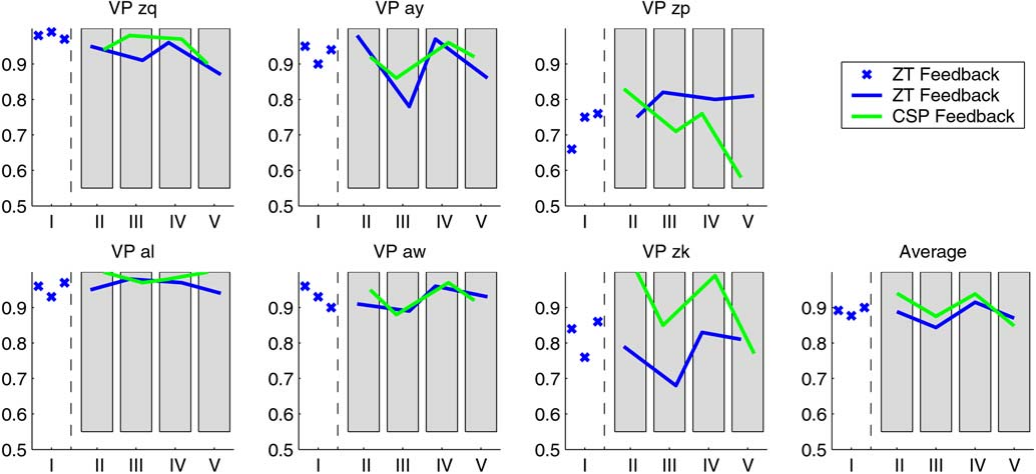
The feedback results for each of the six subjects. The feedback accuracy is denoted for the 100 trials of each run. The initial three runs, here marked as block “I”, were done with the *Zero-Training* classifier, and in the following the order of the classifiers was randomly permuted in each block of two runs, here denoted as “II–V”. The shift of the blue curve relative to the green curve within the shaded areas indicates the order of the classifiers within the block.

The first three runs of feedback showed that all subjects under study were able to operate the BCI with the pre-computed classifier at a high accuracy (only 10 trials per class from the current day were required to update the bias for the classification scenario). For every subject Fig. 4 shows the percentage of successful (“hit”) trials from each run. After the third run, the subjects could not know in advance, which one of the two classifiers (*Zero-Training* or ordinary CSP) was used for the generation of the feedback.

For subjects *zq*, *al* and *zk*, the CSP feedback performed better than the *Zero-Training* feedback. In *ay* and *aw*, the feedback performance on the four blocks is very similar with both classifiers, whereas in subject *zp*, the *Zero-Training* feedback even outperformed the CSP feedback.

**Figure 5:**
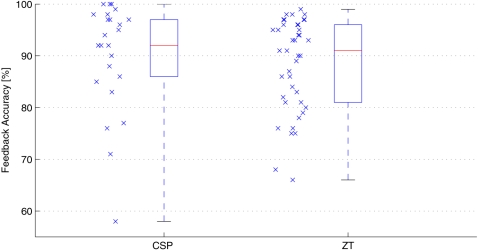
This figure shows the feedback performance of the CSP and the *Zero-Training* classifier over all subjects and runs. The median of the CSP feedback accuracy is slightly higher. This difference is not significant (Wilcoxon ranking test, *p*<0.05).

The performance over all subjects is shown in Fig. 5, where the feedback performance in each run of the four blocks is collected in a single boxplot for each classifier. The CSP performance is slightly higher on average, although this difference is not significant: a Wilcoxon ranking test yields a significance level of *p* = 0.05.

### B. Adaptation of the Classifier Bias

**Figure 6:**
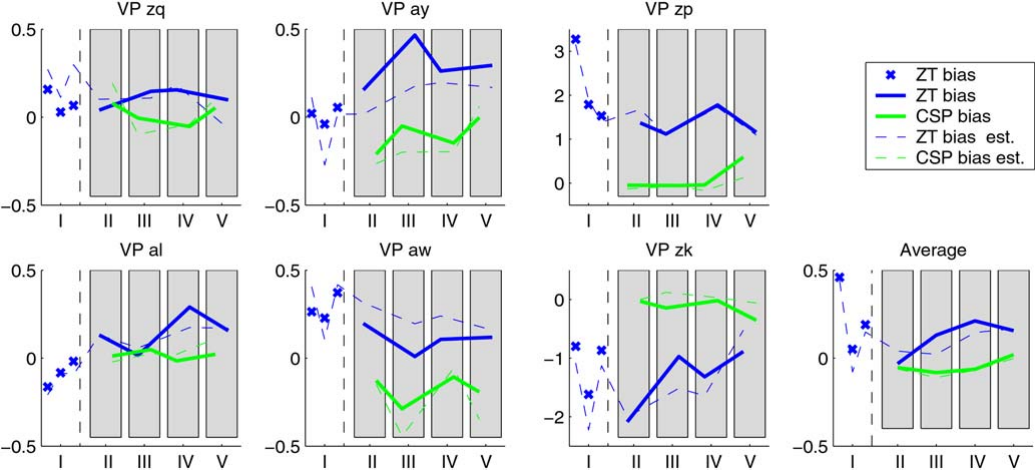
At the beginning of each run, the bias for the classifier was adapted using 10 trials per movement imagination class. The plot shows the optimal bias update, as calculated on the following 100 trials. This value is normalized by the difference between the classifier output class means. The solid lines show the optimal bias for CSP (green) and *Zero-Training* (blue) classifier separately. The dashed lines indicate the bias, as it was actually calculated on the initial 20 trials by the adaptation procedure during the feedback.

The bias was updated at the beginning of every run. We can now check if this update was necessary for the accuracy of the classifiers. For run *i* and classifier *j* and movement class *k*, let μ*_ijk_* be the mean of the classifier output of the corresponding 50 trials. Then the value 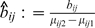 relates the optimal bias *b_ij_* for run *i* and classifier *j* with the actual distance between the class means. A value of 1 would correspond to shifting the decision boundary by the entire inter-means distance. The results of this calculation are shown in Fig. 6. For most subjects, the required shift is moderate (*bˆ* *_ij_*<0.5), but for subjects *zp* and *zk*, the *Zero-Training* classifier requires a strong update of the bias, since the absolute values exceed 1. The CSP classifier, trained on data from the same day, is not as susceptible to bias shift as the *Zero-Training* classifier, since the change is comparatively small also for these two subjects. This finding supports the initial hypothesis that a bias-shift is required for classifiers that are trained on calibration data without visual feedback (such as the *Zero-Training* classifier), whereas the shift *within* the session is comparatively smaller. The latter is the case for the CSP-classifier which is trained on online BCI data with visual feedback. Besides the check for necessity of the bias update, Fig. 6 also provides a comparison of the “optimal” bias with the actual bias, both calculated with the same normalization. The dashed lines indicate the bias, as it was computed on the initial 20 trials during the feedback. From this figure, it is evident that the estimated and the optimal bias coincide quite well. Although the estimation error is sometimes not negligible (as for subjects *aw* and *zk*), the dashed and the corresponding solid lines are highly correlated. If the classifier had not been adapted (corresponding to setting the bias to 0 in Fig. 6), the error would have been larger than is was with the proposed adaptation strategy in nearly all runs. This proves that the update procedure is in fact stable and useful in combination with the *Zero-Training* classifier.

**Figure 7:**
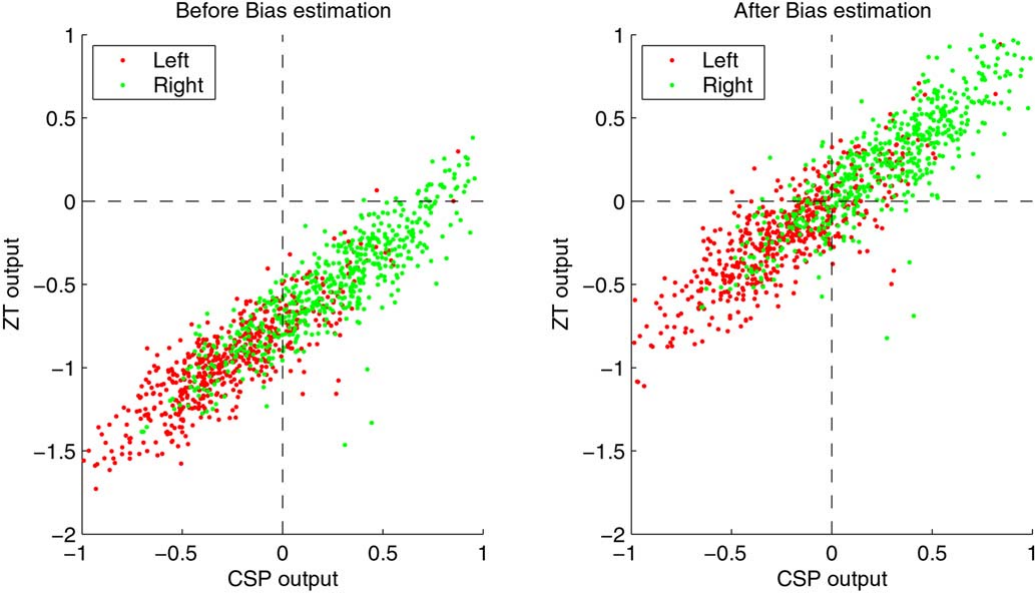
The effect of the bias estimation for subject *zp* (see text for discussion). In the left part of the figure, both *Zero-Training* and the original CSP-classifier are computed on the 1100 trials of the feedback session, without adding a bias term. While the CSP method performs already quite well, the output of *Zero-Training* (on the y-axis) is negative for almost all samples, which would correspond to a classification error near 50%. The right part of the figure shows the output on the same trials, after an initial bias adaptation on the 20 initial trials per run. For the CSP classification, the bias is not changing the result significantly, but *Zero-Training* clearly profits from the bias update.

Fig. 7 exemplifies the effect of the bias shift for subject *zp*. In the left part, the classifiers are calculated for each of the 1100 trials of the feedback, without adding any bias term. While CSP classification (on the x-axis) shows a good separability of the data into positive and negative values (for right hand and left hand movement, respectively), the *Zero-Training* classifier assigns negative values to almost every point, resulting in a poor classification rate (near 50%, corresponding to chance level accuracy). This effect can be alleviated by estimating the bias on the 20 initial trials that were performed previous to every run. The right part of the figure shows the result: both CSP and *Zero-Training* classification rate now are comparable. Note that an improvement of classification accuracy by bias adaptation was highly significant for two subjects.

### C. Discriminability owed to Each Prototype Filter

**Figure 8:**
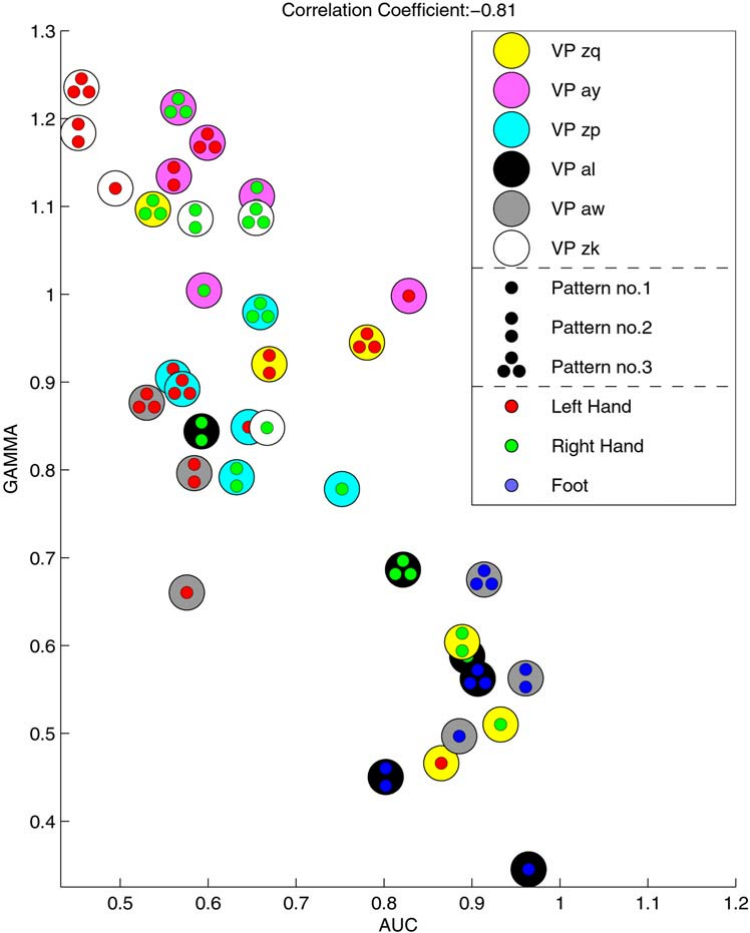
This figure compares the γ-index of a prototypical CSP filter, as calculated on previous sessions, with the discriminability of this feature in the feedback session. The filters with the lowest γ-index have the highest performance. This correlation is highly significant (*p*<0.01).

Here we investigate each prototype CSP filter with respect to the discriminability of the corresponding log-variance feature and relate it to its γ-index, see Section “Prototype Filters”. For the evaluation of the discriminability of each feature, we use as measure the area under the ROC-curve (AUC, see e.g. [24]). This value is 0.5 for features that are uncorrelated with the class affiliation and 1 for features that are perfectly separated. We regarded the γ-index, calculated on the previous sessions, as a prediction of the performance of the feature in the online application of the classifier. Fig. 8 confirms this hypothesis by showing that there is in fact a strong negative correlation between the γ-index and the AUC-value of the features. The higher the density of the CSP filters, accumulated over many sessions, at a particular point, the higher the discriminability of the corresponding log-variance feature in the current online session. Note that below a γ-value of 0.7, only features of the three subjects with the overall highest feedback performances (subjects *al*, *zq* and *aw*) can be found. These features, on the other hand, have the highest AUC-values.

## Discussion

The final validation of BCI algorithms can only be provided in online experiments. However, in contrast to offline evaluation, only one classifier can be applied to the same data set. This makes a comparison especially hard, since the differences between data sets (high inter-subject and inter-session variability) add to the variability of the performance. Therefore it is required to record all data sets under similar conditions. All presented online experiments for one subject were therefore carried out on the same day, which clearly limits the possible number of runs that could be performed. We evaluated the performance of our new classifier by comparing it to the standard CSP method that is typically used for the classification of band power features in motor imagery paradigms (see e.g. [3]). In order to keep the subjects equally motivated under both conditions, we changed the classifier options randomly between runs, but did not inform subjects about the currently used classifier. They were instructed to keep trying to hit the targets given on the screen, irrespective of the possibly fluctuating performance.

The aim of this study was to construct and evaluate a classification method that can be applied without a lengthy calibration measurement. While the features we chose have proven to be quite discriminative for the classification task at hand, the bias adaptation was indispensable for two of the six subjects (and did not degrade the performance for the other subjects). Possible explanations for the shift of the bias from one session to another include the differences in electrode impedances as well as physiological effects like superimposed occipital α-rhythm, see [18], [17], [25]. The number of trials per class used for the adaptation period has to be chosen according to a trade-off between the total duration of the adaptation period and the precision of the bias estimation. After preliminary off-line evaluations we found 10 trials per class to be a quite balanced choice. Note that this number might as well be adjusted according to the predicted feedback accuracy for the subject. Bias parameter estimation is clearly expected to degrade with stronger feedback variance during the adaptation period, and our findings support this expectation. Therefore, if a low feedback performance is expected for a subject, one can easily increase the number of trials used for adaptation. On the other hand the total duration of the adaptation period should be kept very short, since it is desirable for a real-world BCI application to operate right from the start. In such a situation knowledge about class labels is not available and even the equal probability for the occurrence of class labels is not always a reasonable assumption.

**Figure 9:**
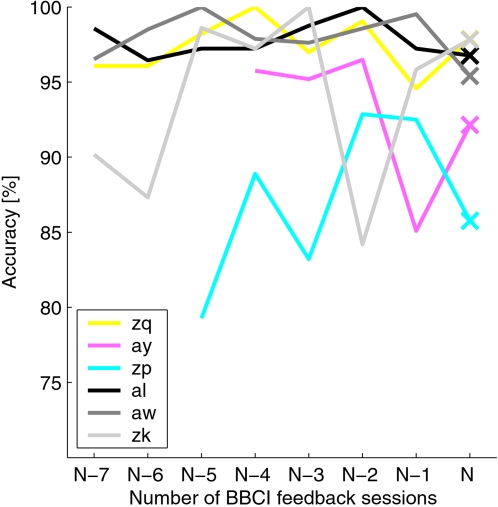
The discriminability of the calibration data for each previous session (*N*−7,…,*N*−1) as calculated by the cross-validation error of the CSP algorithm. Frequency band and time window were specifically optimized for each session and each subject. The cross-validation error on session *N* is calculated on the three runs from block I, with the settings shown in table 1.

In this study, the training data for the CSP-classifier are different from the usual calibration data: in the standard case, no feedback is given during the presentation of stimuli. Also, the visual scene now resembles more closely the feedback setup (see [18]), i.e., the targets are on the left and right side of the screen and they change their color to indicate the next movement task. Although one might suspect that this could degrade the classification performance of the CSP classifier due to the higher complexity of the mental task, this is not the case. Fig. 9 shows the development of the cross-validation error over the previous experiments for each subject. Parameters like the frequency band and the time interval were optimized specifically for each subject and each session. The last point (session *N*) denotes the online experiment performed for this study, where the first three feedback runs were taken into account for training. This corresponds to the data on which the CSP classifier was trained. The cross-validation performance for this session is on the same level as the previous performance and hence does not reveal a systematic disadvantage for the CSP method. On the contrary, the following application of the classifier might even benefit from the fact that the task difference between the training data and the test data is relatively small, as both task have an increased visual complexity.

### A. About Dimensionality

The *Zero-Training* method and standard CSP were used with different data dimensions (12 and 4 resp.). While the absolute numbers might not be very relevant, we would like to explain the motivations that lead to this rather unequal choice: For the standard CSP method, it seems reasonable to expect that it results with spatial filters that are well-adapted to the data of the current session. This is a good argument for fixing the number of spatial filters (and thus the data dimension) to a smaller number. The *Zero-Training* method on the other hand might is dependent on a richer and more robust basis of spatial filters, as the current session might differ from some or most of the historic sessions. Providing the new method with altogether 12 historic filters enhances the probability that one of them is informative also for the new session. It is an interesting open question, whether the larger basis for *Zero-Training* biases the comparison with a systematic disadvantage for the standard CSP method. To investigate, whether standard CSP can profit from a richer basis using more filters, we conducted an offline comparison of classification performance. Here the *Zero-Training* method was fixed to 12 dimensions and compared to standard CSP method with varying dimensions between 4 and 12. However, the offline performance of standard CSP (as a variable of CSP's dimensionality) showed only little variation. It was in the same range as the systematic variance induced by the error estimation technique itself during different cross-validation folds. Given the performance of *Zero-Training* and CSP at eye level end (as shown in Section “Results”) and furthermore that standard CSP can not profit from enlarged dimensionality, this argues in favour of the robust design of our new *Zero-Training* method.

### B. Online Performance and Quality of Adaptation

It has been shown in recent publications [26], [10], that the optimization of spatial and temporal parameters can result in a significantly increased classification accuracy. For the training of the *Zero-Training* classifier however, some of the parameters were not specifically optimized, such as the frequency band, the training window for parameter estimation on the previous sessions, and the movement type combination used for the feedback. These parameters were fixed beforehand. In contrary to this, the subject-dependent parameters of the standard CSP method were selected individually based on the same day's training data. We are fully aware, that this comparison strategy may have resulted in a slight advantage in favor of the standard CSP method, but we accepted this advantage in order to have a maximally strong adversary method available for the comparison with our new *Zero-Training* method.

Only in subject *zk*, the CSP classifier clearly outperforms the *Zero-Training* classifier. The reason might be due to the smaller amount of training data which was present for *zk* from previous sessions: while the training sessions for all other subjects contained more than 100 trials per class, only 35 trials per class and session were recorded for subject *zk*, see also table 1. This circumstance leads to a higher variability in the collection of CSP filters and it explains the low γ-index for all features of subject *zk*, see Fig. 8.

**Figure 10:**
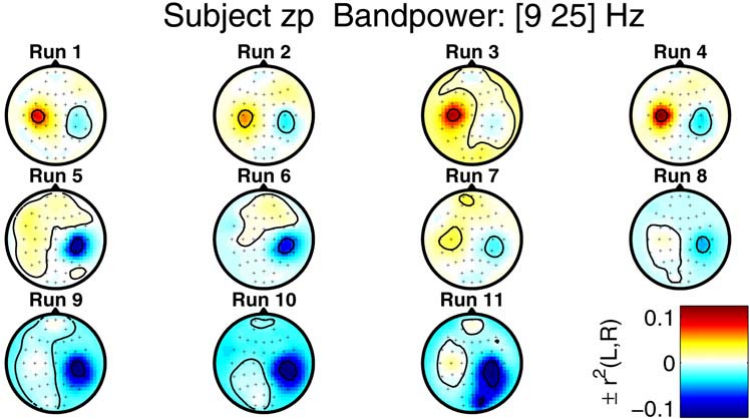
For each feedback run of the session, this figure shows the scalp topographies of class discriminability of band power features for subject *zp*. After bandpass filtering to the frequency band of 10–25 Hz, the log-bandpower was calculated for each electrode in the window 500–3000 ms after the presentation of the stimulus. Finally, signed *r* ^2^-values were calculated as a measure of class discriminability.

For subject *zk*, the γ-values for the *Zero-Training* features are slightly higher than for subject *zp*. From the feedback performance in Fig. 4, we can even see a slow positive trend for the *Zero-Training* classifier throughout the day. The trend in the performance for the CSP classifier, on the other hand, is degrading over time. Subject *zp* reported that she was trying to control the feedback with different strategies over time, always switching to the mental imagery that seemed most reliable at each point in time. This variability in the mental strategies, induced by the feedback presentation, is reflected in the brain signals. Fig. 10 shows the evolution of the scalp topographies related to the discriminability of the band power features in each electrode. We calculated the band power features for the 100 feedback trials in each run and calculated the *r* ^2^-values between left and right hand imagery class, as a measure of linear discriminability. The figure shows that towards the end of the session, the features on the right motor cortex are more discriminative than the features initially on the left motor cortex. The feedback performance of the CSP classifier appears to be more susceptible to this shift, while the *Zero-Training* classifier is based on a broader basis of spatial filters, which can account for this variability in the signals.

### C. Long-term variability

BCI performance is known to vary on multiple time scales. The quality of the presented new classification approach can only be rated in terms of its immediate applicability when the subjects are confronted with the specific feedback for the first time. By limiting the experimental sessions to a single day for each subject, we ensured that no long-term training effects can bias our experimental results. On the other hand, significant positive trends can be observed for static classifier setups when comparing performance across sessions, see e.g. [27]. Hence, we expect that the reiterated use of our classifiers on further experimental sessions will lead to results of a similar or even higher quality.

Although the complex interaction process of subjects' long-term adaptivity with this classifier is beyond the scope of this paper, note that this setup contains a largely static classifier based on specific brain signatures of particular subjects. It can be regarded as a promising starting point for further performance enhancements by operant conditioning.

### D. Adaptive Classification

A possible remedy for the degrading performance is the adaptive estimation of the linear hyperplane of the classifiers, [17], [28]. Using an adaptation period as short as 10 trials per class, however, the adaptation of the hyperplane for *Zero-Training* fails for almost every subject, as an offline evaluation on the given data shows. This is mainly due to the fact that for a linear classifier, the number of parameters to be estimated grows quadratically with the number of feature dimensions. Since the *Zero-Training* feature space has 12 dimensions (6 “prototype” filters and 6 “CSP” filters), 20 trials are too few data. Similar results have been shown in [17] for classical CSP; the suggested bias update requires only the estimation of one single parameter and is therefore more robust. If, however, the feature discrimination performance is changing over time like in subject *zp*, this bias update might not be sufficient any more. Other options, like a continuous adaptation of the bias throughout the feedback run, require at least the a posteriori knowledge of all the labels of this run, which can not be granted in all feedback applications. Moreover, this continuous adaptation scheme did not prove to be superior to the initial adaptation of the bias [18].

Fig. 8 suggests a good prediction accuracy for prototypical CSP filters with a low γ-index. However, since the features of some subjects (e.g. *zk* and *zp*) appear to form distinct clusters for each class, we should consider some reasonable normalization between these values. The γ-index, as formulated above, depends mainly on the number of dimensions and on the number of samples. This holds true because the maximally possible γ-index is a monotonic decreasing function in the number of samples, if the number of dimensions (in this case: the number of electrodes) is fixed. Not only the maximal, but also the expected minimal γ-index under randomly drawn samples will differ. Therefore, we estimated this value by a simulation: the number of dimensions and samples were chosen for every subject according to Table 1. The minimal γ-value was calculated and averaged over 1000 repetitions. The results are displayed in Table 2. Since the values range from 1.12 for subject *aw* to 1.22 for subject *ay*, the correlation visualized in Fig. 8 is not influenced under the condition, that each γ-value is normalized by the expected minimal γ-value. Note that for subjects *zk* and *ay*, some of the γ-values are close to 1 after normalization; this corresponds to a minimal “cluster” density which is expected to occur even in random samples. As expected, these features have only very low AUC-values.

**Table 2:** This table shows the minimal γ-index for a collection of randomly drawn points, together with the standard deviation.

Subject	Expected Minimal γ
*zq*	1.17±0.02
*ay*	1.22±0.02
*zp*	1.20±0.02
*al*	1.15±0.02
*aw*	1.12±0.02
*zk*	1.17±0.02

For this calculation, the same dimensionality (corresponding to the number of electrodes) and the same number of points (corresponding to three times the number of experiments) was used.

With respect to the cumbersome electrode preparation great advancements could be achieved in the meantime by newly developed hardware. In [4] we present a novel dry EEG recording technology which does not need preparation with a conductive gel. The study with good BCI subjects revealed that the feedback performance using the new sensor technology was comparable to the approach with conventional EEG caps for most subjects. Note that the system reported in [4] only uses 6 electrodes and can thus be miniaturized to run with a tiny EEG amplifier and a pocket PC.

### E. Conclusion

Brain-Computer Interfacing has seen a rapid development in the recent years. A main step forward towards a broader usability of this technology even beyond rehabilitation was the drastic reduction of user training from 60–150 hours of subject training to less than 30 minutes of calibration [29], [2]. The latter became possible by virtue of modern machine learning methods for BCI [30], [31].

In this contribution we went one step further towards the goal of avoiding subject training altogether and proposed novel algorithms to transfer knowledge between BCI sessions. Our study shows that the results from prior off-line analysis, successfully carry over to the present set of online experiments, where subjects use decoders that were constructed from past data instead of calibrating anew. Our findings thus show that information from prior session can indeed be used profitably for constructing better individual mental state decoders. Note that the loss in performance (bitrate) is negligible when contrasted to employing a fully calibrated decoder (after 30 minutes of training) in a blind protocol.

Our work opens therefore a highly promising path for the ultimate goal of *Zero-training*. While the proposed methods work well for session to session transfer for an individual subject, it remains still open, whether inter-subject information could also be successfully transferred. Ideally a data base consisting of individualized decoders could be appropriately combined as an ensemble decoder and thus help to avoid training completely. In combination with dry electrodes [4], *Zero-training* would again provide a large step forward when striving towards more general applicability of BCI technology for daily use in man machine interaction.
